# Examining the mediating role of muscle quantity in adolescents: associations with adiposity, cardiorespiratory fitness, muscular fitness, and cardiometabolic risk factors

**DOI:** 10.1038/s41598-024-61805-w

**Published:** 2024-05-26

**Authors:** Neiva Leite, Maiara C. Tadiotto, Frederico B. de Moraes Junior, Francisco J. de Menezes-Junior, Patricia R. P. Corazza, Larissa R. da Silva, Wendell A. Lopes, Oslei de Matos, Rosana B. Radominski, Manuel J. Coelho-e-Silva

**Affiliations:** 1 Department of Physical Education, Federal University of Paraná, Street Col. Francisco H. Dos Santos, 100, Jardim das Americas, Curitiba, Paraná 81531-980 Brazil; 2 State University of Western Paraná, Cascavel, Brazil; 3 State University of Maringá, Maringá, Brazil; 4 Technological Federal University of Paraná, Curitiba, Brazil; 5 University of Coimbra, FCDEF, Coimbra, Portugal; 6 University of Coimbra, CIDAF (Uid/04213/2020), Coimbra, Portugal

**Keywords:** DXA, Physical fitness, Low muscle mass, Lean mass index, Muscle-to-fat ratio, Biomarkers, Endocrinology, Health care, Risk factors

## Abstract

The aim of this study was to evaluate the mediation role of muscle quantity in the relationship between physical fitness and cardiometabolic risk factors (CMRF) in adolescents. This cross-sectional study conducted with 120 adolescents of both sexes, aged between 10 and 17 years. Body mass, height, fat mass (FM), lean mass, blood pressure, high-density lipoprotein, low-density lipoprotein, triglycerides, glucose, insulin, cardiorespiratory fitness (CRF) and 1 repetition maximum strength (1-RM) with evaluation of the leg press 45° (RM-leg), bench press (RM-bench) and arm curl (RM-arm). Body mass index z-score, appendicular skeletal muscle mass, appendicular skeletal muscle mass index, lean mass index (LMI), muscle-to-fat ratio (MFR), age at peak height velocity, and CMRF z-score were calculated. The direct relation between FM and CMRF was mediated by the LMI (26%) and inverse relation between CRF and CMRF was mediated by the LMI (26%). For girls, the direct relation between FM and CMRF was mediated by the LMI (32%); the inverse relation between CRF, RM-leg, RM-arm and CMRF was mediated by the LMI (32%, 33%, and 32%, respective). For boys, the indirect effect was not significant, indicating that LMI is not a mediator in the relation between FM, CRF, 1-RM with CMRF. The direct relation between RM-leg and CMRF was mediated by the MRF (16%). This finding evidenced the importance of promoting a healthy lifestyle to improve physical fitness levels and the quantity of muscle mass in adolescents.

## Introduction

Changes in the quantity and quality of muscle mass can occur due to increased sedentary behavior and the presence of obesity^1,2^, factors that reduced muscular fitness, cardiorespiratory fitness (CRF), physical performance, mobility, and quality of life^1–4^. Furthermore, the aging process is responsible for changes in muscle quantity (reduction in fiber number and muscle cross-sectional area) and changes in muscle quality, responsible for increases in both extra muscular and intramuscular fat^5,6^. Adolescence is a period of somatic and biological growth and the transition from adolescence to adulthood is associated with changes in body composition, with an increase in muscle mass and change in body fat distribution^1^. Thus, the adequate relationship between the amount of muscle mass and fat mass for metabolic health is recognized, with an important role in protein metabolism, insulin sensitivity, glucose, and fatty acid oxidation, suggesting that factors supporting muscle quality may also be determinants of obesity-associated morbidity^3,7^. However, size and mass are not the only physiological attributes of muscle in relation to health and disease. Obesity-related changes in muscle composition (that is, muscle quantity and quality) may be of clinical importance, as ectopic fat accumulation in muscle has been reported in children with obesity^2^.

In children and adolescents, decreases in health-related components of physical fitness impair daily activities and are associated with increased mortality in adults and older adults^8^. CRF stands out as an important marker of health status in adults and youth^9,10^, whose performance is probably associated with muscle strength. In general, low muscle mass and low CRF, can compromise and impair motor competence in adolescents with obesity^11^, reducing mobility and agility of children and adolescents^12^, as well as less participation in spontaneous physical activities^13^, especially in presence of body weight overload^14^. Therefore, functional impairments in people with obesity can make it difficult to perform daily activities and less engagement in regular physical exercise practices, resulting in lower muscle quantity and quality.

Thus, there is the potentiation of muscle changes arising from chronic diseases and not from the aging process, some authors have termed as secondary sarcopenia^15^ or sarcopenic obesity^16^. In adults and the elderly, the diagnosis can be made by the decrease in physical capacity or muscle strength associated with low skeletal muscle mass. The greatest concern is the repercussions of sarcopenic obesity in the elderly^17^, but there are indications that it starts earlier, in the youth phase^18^, due to the inflammatory process associated with obesity. In a systematic review, the authors conclude that there is a need to develop a definition and standardize assessment in pediatric population, as well, they suggest carrying out studies to understand the relationship between obesity and sarcopenia, and their association with health problems in children and adolescents^18^.

Researchers propose the assessment of muscle strength by handgrip, with different cut-off points according to age group^19^, as well as using the one repetition maximum test^20^. Moreover, there is a proposal to analyze the severity of the disproportionate relationship between fat and lean tissues by the ratio between the muscle mass and fat mass or muscle-to-fat ratio (MFR), used as an indicator of cardiometabolic risk^21^. There is an inverse relation between muscle mass and the onset of cardiometabolic diseases^21^. Decreased muscle mass reduces basal metabolic expenditure and favors increased adiposity, worsening insulin resistance^1^ and increasing the cardiometabolic risk^8^. However, it is not clear whether there is a synergism between the action of obesity and disproportionate ratio of fat and lean mass in the worsening of the clinical metabolic situation, when compared to sarcopenia or obesity alone in adults^22^.

In children and adolescents, some authors highlighted the reduction of muscle mass in the relation to excessive fat mass^23^, as well as the "Fat but Fit" interaction between adiposity and physical fitness, which suggests that fitness may attenuate the deleterious effects of excess weight^24^. Therefore, the vicious cycle of reduced muscle mass and increased adiposity contribute to lower relative muscle mass also in adolescents, which can potentiate the inadequate metabolic profile^25^, as well as the early onset of hypertensive measures^26^. Recent studies focus on reduction in muscle quantity and cardiometabolic alterations^16,23^, as well as understanding the mediating role of muscle quantity in relation between adiposity and bone mineral content^27^. However, there is a scientific gap on the mediating role of muscle quantity, according to adiposity, components of physical fitness and the presence of cardiometabolic factors in this age group. Our hypothesis is that muscle quantity mediates the relationships between adiposity, physical fitness and cardiometabolic risk factors in adolescents. Thus, the aim of this study was to evaluate the mediation role of muscle quantity in the relationship between physical fitness and cardiometabolic risk in adolescents.

## Methods

### Ethics and inclusion statement

The present study was approved by the Research Ethics Committee of the UniDBSCO University Center (CAEE: 62963916.0.0000.5223) and was carried in accordance with the norms of Resolution 196/96 of the National Health Council on research involving human beings in Brazil.

### Study design and population

This is a cross-sectional study with a correlational descriptive character. The population consisted of adolescents from Curitiba and the Metropolitan Region—Paraná/Brazil. The recruitment was conducted in a non-probabilistic sampling process, for convenience. Adolescents, parents and/or guardians were informed about the research procedures and signed informed consent form. Data collection was conducted between March and April 2019.

This study recruited 231 adolescents and included 120 adolescents of both sexes, aged between 10 and 17 years, who participated in all assessments. Sample size was calculated a priori using the G*Power software, through the linear regression, with four predictive variables. Power of 0.90, an alpha of 0.05, and effect size (f) of 0.15 were assigned. Based on these criteria, the minimum sample size was 108 participants. The inclusion criteria were: (a) participate in all assessments; (b) no present contraindications to the tests, including the absence of heart, pulmonary and osteoarticular diseases; and (c) not using drugs that interfere with research results.

### Anthropometric measures

Measurements were performed according to the procedures described in the literature^28^. Height was measured using a portable stadiometer (Avanutri®) with an accuracy of 0.1 cm, and body mass was measured using a digital reading platform scale (Welmy®), with a maximum capacity of 200 kg and an accuracy of 0.1 kg. Body mass index z-score (BMI-z) were calculated in the WHO Anthro Plus®^29^. Waist circumference measurement was evaluated with a flexible and inextensible tape with a resolution of 0.1 cm (Sanny®)^28^.

### Dual energy X-ray absorptiometry

For the analysis of body composition, dual energy x-ray absorptiometry (DXA) was performed with a Hologic QDR® model 4500 device and the information was analyzed by the equipment software. For the examination, the adolescents wore appropriate clothing and were asked to remove any jewelry or metallic accessory from the body that could interfere. After, they should down in dorsal decubitus on the equipment table. Scanning was conducted from the upper region of the skull to the ends of the toes and lasted approximately two to five minutes. Fat mass, lean mass, lean arms mass, lean legs mass, were determined with a resolution of 1 g and described in kg.

### Muscle quantity

Appendicular skeletal muscle mass (ASM) was calculated as the sum of lean mass from arms and legs (kg). Appendicular skeletal muscle mass index (ASMI) was calculated as the ASM divided by height squared (kg m^−2^)^30^. Lean mass index (LMI) was calculated as lean mass divided by height squared (kg m^−2^)^31^. Muscle-to-fat ratio (MFR) was calculated as total lean mass divided by total fat mass (kg)^19^.

### Clinics and metabolic variables

Systolic blood pressure (SBP) and diastolic blood pressure (DBP) were measured using a previously calibrated mercury sphygmomanometer, with the appropriate cuff size for the arm circumference, with the adolescent sitting and right arm supported at heart level, after resting for ten minutes. Two measurements were performed at a 1-min interval, with the lowest value being considered^32^. Blood samples were collected in the morning by specialists using standard techniques after twelve hours of fasting. The colorimetric enzymatic method was used to measure high-density lipoprotein cholesterol (HDL-c), low-density lipoprotein cholesterol (LDL-c), triglycerides (TG) and insulin. The chemiluminescence method was used to determine glucose. Homeostasis metabolic assessment insulin resistance (HOMA-IR)^33^ and quantitative insulin sensitivity check index (QUICKI)^34^ were determined. Cardiometabolic risk factors (CMRF) score was calculated (z-score): SBP + DBP + HDL-c + LDL-c + TG + HOMA-IR. HDL-c was multiplied by ^−1^. This approach was used in similar studies^35,36^.

### Cardiorespiratory fitness

Peak oxygen consumption (VO_2peak_) was assessed using the maximum incremental test, on a treadmill, using a K4b^2^ metabolic analyzer (Cosmed®). A warm-up was performed with a five-minute walk of 2.7 km h^−1^. Protocol started at a speed of 4.0 km h^−1^, a progressive increase of 0.6 km h^−1^ every minute and a constant and fixed inclination of 1%. After the test is completed, a walk at 5.0 km h^−1^ was performed, reduction of 1.0 km h^−1^ every minute for three minutes. A heart rate monitor (Polar®) was used to determine the maximum heart rate (HR_max_). Test was considered maximum when two of the criteria were observed: (a) exhaustion or inability to maintain the required speed^37^; (b) respiratory exchange ratio ≥ 1.0; (c) achieving the HR_max_ predicted^38^.

The VO_2peak_ was determined after filtering in 15 s intervals and identified by the highest value obtained after the VO_2_ plateau. Values of VO_2_ increase < 50 mL/min^−1^ in the last 30 s of the stress tests were interpreted as a VO_2_ plateau. VO_2peak_ was expressed in absolute units (L min^−1^) and relative to body mass (mL kg min^−1^). All assessments were carried out by two trained evaluators in a controlled environment in which the ambient temperature varied between 20 and 25 °C and the relative humidity between 40 and 60%.

### Muscular fitness

Maximum strength was measured by the one-repetition maximum test (RM)^20^. Exercises used were leg press 45° (RM-leg press), bench press (RM-bench press) and arm curl (RM-arm curl), performed in three sessions, with an interval of 48 h between each session. In session 1, familiarization was performed, with three sets of eight to ten repetitions with progressive loads to reach approximately 50% of the load. Session 2, a series of eight repetitions began with approximately 50% of the perceived load in session 1. After a one-minute of rest, the second series of three repetitions was performed at 70% of the perceived load, and after two minutes of rest, were submitted to the RM test with 100% of the perceived load. If successfully executed, 5–10% of the load was added until reaching the maximum load with a rest of three to five minutes. When not successfully executed, it reduced 50% of the increment of the last attempt. In session 3, the test followed the same procedure as in session 2, with the aim of confirming the previous load or carrying out new increments, if necessary. The highest load achieved between sessions 2 and 3 was considered for analysis.

### Covariates

Sex, chronological age, somatic maturation, and fat mass are considered covariates. Adolescence is a period of somatic and biological growth, and so that these changes do not confound the results, the analyses were controlled considering somatic maturation. Somatic maturation was estimated by determining the distance in years from peak height velocity by the mathematical model based on height, age, and sex. Prediction of age at peak height velocity was determined by subtracting the maturity offset from the chronological age (years)^39^.

### Statistical analysis

Shapiro–Wilk test was used to verify the normality of the data. Standard descriptive statistics were used to characterize the sample. For analysis purposes, three groups were defined by BMI-z: Eutrophic (score < 1), Overweight (between score ≥ 1 and < 2) and Obese (score ≥ 2). Comparisons between groups were verified by one-way analysis of variance with Bonferroni's post-hoc for parametric variables and the Kruskal–Wallis for non-parametric. Spearman's correlation coefficient was used to verify relationships.

Mediation analysis was determined from linear regression models (bootstrapping procedures, 10,000 re-samplings; 95% IC BCa). This approach considered muscle quantity independent variable, CRF and muscular fitness as mediators, and cardiometabolic risk factors as dependent variables. Mediation models were constituted: equation (a) independent variable by the mediator; equation (b) mediator by the dependent variable; equation (c) independent variable by the dependent variable (total effect); and equation (c′) independent variable and mediator by the dependent variable (direct effect). The indirect effect was estimated by ab equations, and it was significant if zero was outside the 95% CI^40^. The percentage of mediation was estimated as 1 − (c′/c). Analyzes were adjusted for sex, age, age at peak height velocity and fat mass. Data analysis was performed with the SPSS v.24.0, the moderation models in the PROCESS macro for the SPSS, and the significance level was established at p ≤ 0.05.

### Ethics approval

The study protocols has been approved by the Ethics Committee of the UniDBSCO University Center—Brazil (n. 1.990.654, CAAE 62,963,916.0.0000.5223/2017).

### Consent to participate

Parents and/or guardians and adolescents signed the terms of consent for participation in the research.

### Informed consent

Informed consent was obtained from all participating teams in the study.

## Results

Descriptive characteristics of the adolescents separated by sex and allocated by BMI-z are summarized in Table 1. In the total sample, age, MFR and QUICKI were higher in eutrophic, while fat mass, lean mass, LMI, systolic blood pressure, triglycerides, insulin, HOMA-IR, and RM-leg press were higher in obese. No differences were found for the other variables (data not presented). For boys, age, MFR and QUICKI were higher in eutrophic, while waist circumference, fat mass, LMI, insulin, HOMA-IR were higher in obese. For girls, MFR and QUICKI were higher in eutrophic, while waist circumference, fat mass, lean mass, ASMI, LMI, insulin, HOMA-IR, VO_2peak_, RM-leg press, and RM-arm curl were higher in obese. In the total sample, the CRMF was associated with muscle quantity and physical fitness, except RM-bench press. For boys, the CMRF was only associated with LMI and MRF. For girls, the CMRF was associated with ASM, LMI, and MRF, as well as VO_2peak_, RM-leg press and RM-arm curl (Table 2).

**Table 1:** Descriptive characteristics (mean ± standard deviation) of the adolescents separated by sex (boys and girls) and BMI-z.

Variables	Boys (n = 52)				Girls (n = 68)			
	Eutrophic (n = 13)	Overweight (n = 12)	Obese (n = 27)	p	Eutrophic (n = 17)	Overweight (n = 22)	Obese (n = 29)	p
Age (years)^ǂ^	15.3 ± 1.0^a^	15.1 ± 1.3	13.8 ± 1.8	**0.018**	15.4 ± 1.1	14.3 ± 1.6	14.6 ± 1.6	0.099
Age PHV (years)^ǂ^	13.7 ± 0.4	13.7 ± 0.5	13.4 ± 0.6	0.055	12.5 ± 0.3	12.4 ± 0.5	12.3 ± 0.5	0.258
Height (cm)^ǂ^	173.0 ± 5.7	171.5 ± 5.7	168.0 ± 10.8	0.427	162.8 ± 4.1	158.9 ± 7.7	162.1 ± 6.8	0.097
Body composition and muscle quality								
Body mass (kg)^ǂ^	59.9 ± 5.5^a^	73.8 ± 9.7^a^	86.6 ± 18.6	** < 0.001**	56.1 ± 5.0^a,b^	64.5 ± 9.1^a^	80.6 ± 11.7	** < 0.001**
Waist circumference (cm)	70.2 ± 5.7^a,b^	82.4 ± 6.5^a^	93.4 ± 9.3	** < 0.001**	68.1 ± 4.4^a,b^	77.5 ± 5.3^a^	87.1 ± 6.8	** < 0.001**
Fat mass (kg)	11.4 ± 8.6^a,b^	20.7 ± 5.3^a^	34.3 ± 10.1	** < 0.001**	17.2 ± 5.0^a,b^	25.7 ± 5.2^a^	35.8 ± 7.6	** < 0.001**
Lean mass (kg)^ǂ^	47.0 ± 3.9	49.6 ± 5.1	49.4 ± 9.6	0.479	36.3 ± 3.5^a^	36.2 ± 4.6^a^	41.5 ± 5.6	** < 0.001**
ASM (kg)^ǂ^	20.7 ± 1.8	21.8 ± 2.7	20.9 ± 4.7	0.631	14.8 ± 1.7	14.0 ± 1.7	15.8 ± 2.8	0.090
ASMI (kg·m^-2^)^ǂ^	6.9 ± 0.6	7.4 ± 0.6^a^	7.3 ± 1.0	0.154	5.6 ± 0.6	5.5 ± 0.4^a^	6.0 ± 0.7	**0.033**
Lean mass index (kg·m^-2^)	15.7 ± 1.3^a,b^	16.9 ± 1.3^a^	17.3 ± 1.8	**0.003**	13.7 ± 1.1^b^	14.3 ± 1.0^a^	15.8 ± 1.4	** < 0.001**
Muscle-to-fat ratio^ǂ^	5.77 ± 3.1^a,b^	2.55 ± 0.7^a^	1.50 ± 0.4	** < 0.001**	2.39 ± 1.2^a,b^	1.45 ± 0.2^a^	1.20 ± 0.2	** < 0.001**
Clinics and metabolic variables								
Systolic BP (mm Hg)^ǂ^	103.8 ± 11.4	110.5 ± 11.0	111.2 ± 8.8	0.102	99.8 ± 10.9	103.3 ± 14.8	107.5 ± 9.2	0.067
Diastolic BP (mm Hg)^ǂ^	65.5 ± 8.0	63.7 ± 6.8	66.5 ± 9.2	0.679	61.4 ± 7.6	64.8 ± 10.9	66.5 ± 9.2	0.205
HDL-c (mg·dL^-1^)	54.2 ± 10.1	53.1 ± 7.0	50.3 ± 11.1	0.423	55.3 ± 6.2	57.9 ± 13.2	57.8 ± 10.7	0.725
LDL-c (mg·dL^-1^)	72.4 ± 15.8	87.5 ± 20.2	83.1 ± 28.7	0.295	80.4 ± 25.7	81.7 ± 27.4	92.8 ± 26.1	0.171
Triglycerides (mg·dL^-1^)^ǂ^	79.1 ± 24.1	94.6 ± 45.3	109.7 ± 47.5	0.215	95.5 ± 37.9	89.5 ± 34.4	128.5 ± 86.0	0.124
Insulin (µUI·mL^-1^)^ǂ^	7.2 ± 2.6^a,b^	11.7 ± 4.8^a^	17.0 ± 9.3	** < 0.001**	10.5 ± 3.8^b^	11.6 ± 3.8^a^	18.2 ± 10.3	**0.001**
HOMA-IR^ǂ^	1.41 ± 0.5^a,b^	2.41 ± 0.9^a^	3.53 ± 1.9	** < 0.001**	2.23 ± 0.9^b^	2.47 ± 0.9^a^	3.99 ± 2.5	**0.002**
QUICKI	0.368 ± 0.02^a,b^	0.339 ± 0.02^a^	0.324 ± 0.02	** < 0.001**	0.343 ± 0.02^b^	0.337 ± 0.02^a^	0.319 ± 0.02	**0.001**
Cardiorespiratory and muscular fitness								
VO_2peak_ (L min^−1^)	2.82 ± 0.3	3.06 ± 0.6	2.88 ± 0.7	0.633	2.01 ± 0.3^a^	2.13 ± 0.3^a^	2.44 ± 0.3	** < 0.001**
VO_2peak_ (mL kg min^−1^)^ǂ^	47.3 ± 4.7^a^	41.6 ± 5.7^a^	33.5 ± 6.9	** < 0.001**	36.0 ± 6.0^a^	33.3 ± 2.7^a^	30.4 ± 3.7	** < 0.001**
RM-leg press (kg)^ǂ^	184.6 ± 33.7	195.4 ± 56.3	195.2 ± 55.7	0.638	147.3 ± 26.9^b^	146.4 ± 33.2^a^	184.2 ± 33.1	** < 0.001**
RM-bench press (kg)^ǂ^	39.8 ± 11.1	44.3 ± 11.3	37.6 ± 11.9	0.313	28.5 ± 5.3	28.0 ± 6.1^a^	31.4 ± 4.9	0.094
RM-arm curl (kg)^ǂ^	23.1 ± 5.5	24.2 ± 6.4	20.5 ± 5.8	0.238	16.5 ± 3.2	16.0 ± 2.9^a^	18.5 ± 3.1	**0.004**

*PHV* peak height velocity, *ASM* appendicular skeletal muscle mass, *ASMI* appendicular skeletal muscle mass index, *BP* blood pressure, *HDL-c* high density lipoprotein, *LDL-c* low density lipoprotein, *HOMA-IR* homeostasis model to assessment insulin resistance, *QUICKI* quantitative insulin sensitivity check index, *VO*_*2peak*_ peak oxygen consumption, *RM* repetition maximum, ^ǂ^Nonparametric test, ^a^Different from obese, ^b^Different from overweight), bold (significance p ≤ 0.05).

**Table 2:** Correlation between muscle quantity variables, cardiorespiratory fitness, muscular fitness and cardiometabolic risk factors score.

Variables	ASM		ASMI		LMI		MFR		VO_2peak_		RM-leg press		RM-bench press		RM-arm curl	
	rho	p	rho	p	rho	p	rho	p	rho	p	rho	p	rho	p	rho	p
All (n = 120)																
ASM	–		–		–		–		0.820	** < 0.001**	0.598	** < 0.001**	0.743	** < 0.001**	0.721	** < 0.001**
ASMI	–		–		–		–		0.722	** < 0.001**	0.597	** < 0.001**	0.726	** < 0.001**	0.654	** < 0.001**
LMI	–		–		–		–		0.657	** < 0.001**	0.732	** < 0.001**	0.713	** < 0.001**	0.664	** < 0.001**
MFR	–		–		–		–		0.202	**0.027**	0.047	0.610	0.340	** < 0.001**	0.284	**0.002**
WC	0.290	0.001	0.342	** < 0.001**	0.566	** < 0.001**	− 0.584	** < 0.001**	0.365	** < 0.001**	0.434	** < 0.001**	0.203	**0.026**	0.217	**0.017**
FM	0.051	0.581	0.003	0.974	0.226	**0.013**	− 0.856	** < 0.001**	0.191	**0.037**	0.285	**0.002**	0.021	0.819	0.065	0.482
CMRF	0.190	**0.038**	0.204	**0.026**	0.370	** < 0.001**	− 0.296	**0.001**	0.281	**0.002**	0.222	**0.015**	0.134	0.146	0.179	**0.050**
Boys (n = 52)																
ASM	–		–		–		–		0.604	** < 0.001**	0.582	** < 0.001**	0.719	** < 0.001**	0.646	** < 0.001**
ASMI	–		–		–		–		0.500	** < 0.001**	0.650	** < 0.001**	0.755	** < 0.001**	0.652	** < 0.001**
LMI	–		–		–		–		0.410	**0.003**	0.730	** < 0.001**	0.658	** < 0.001**	0.581	** < 0.001**
MFR	–		–		–		–		0.113	0.426	0.012	0.932	0.284	**0.042**	0.342	**0.013**
CMRF	− 0.014	0.920	0.205	0.145	0.374	**0.006**	− 0.417	**0.002**	0.068	0.633	0.150	0.288	0.023	0.871	− 0.022	0.877
Girls (n = 68)																
ASM	–		–		–		–		0.631	** < 0.001**	0.478	** < 0.001**	0.426	** < 0.001**	0.474	** < 0.001**
ASMI	–		–		–		–		0.398	**0.001**	0.427	** < 0.001**	0.371	**0.002**	0.306	**0.011**
LMI	–		–		–		–		0.526	** < 0.001**	0.644	** < 0.001**	0.548	** < 0.001**	0.539	** < 0.001**
MFR	–		–		–		–		− 0.361	**0.002**	− 0.271	**0.026**	− 0.047	0.705	− 0.218	0.075
CMRF	0.246	**0.044**	0.182	0.138	0.377	**0.002**	− 0.376	**0.002**	0.410	**0.001**	0.306	**0.011**	0.232	0.057	0.374	**0.002**

*ASM* appendicular skeletal muscle mass, *ASMI* appendicular skeletal muscle mass index, *LMI* lean mass index, *MFR* muscle-to-fat ratio, *VO*_*2peak*_ peak oxygen consumption, *RM* repetition maximum, *CMRF* cardiometabolic risk factors, bold (significance p ≤ 0.05).

**Figure 1:**
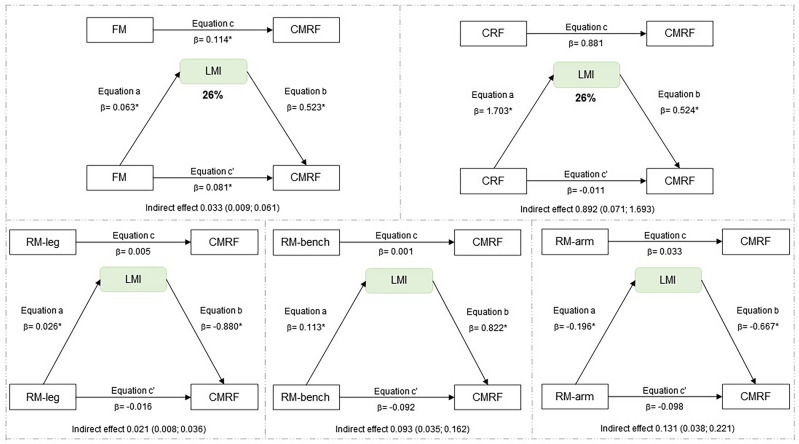
Mediation of lean mass index in the relationship between physical fitness and cardiometabolic risk for the total sample. All analyzes were controlled by sex, age, age at peak height velocity and fat mass. *FM* fat mass, *CRF* cardiorespiratory fitness, *RM-leg* repetition maximum-leg press, *RM-bench* repetition maximum-bench press, *RM-arm* repetition maximum-arm curl, *LMI* lean mass index, *CMRF* cardiometabolic risk factors score, *significance p ≤ 0.05.

**Figure 2:**
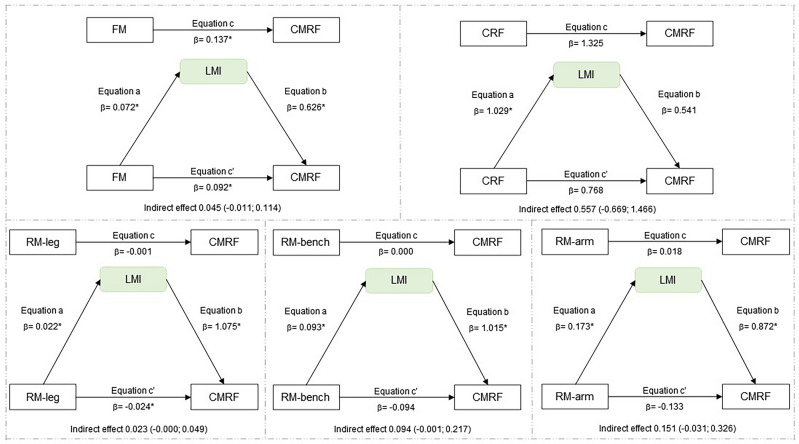
Mediation of lean mass index in the relationship between physical fitness and cardiometabolic risk in boys. Analyzes were controlled by age, age at peak height velocity and fat mass. *CRF* cardiorespiratory fitness, *RM-leg* repetition maximum-leg press, *RM-bench* repetition maximum-bench press, *RM-arm* repetition maximum-arm curl, *LMI* lean mass index, *CMRF* cardiometabolic risk factors score, *Significance p ≤ 0.05.

**Figure 3:**
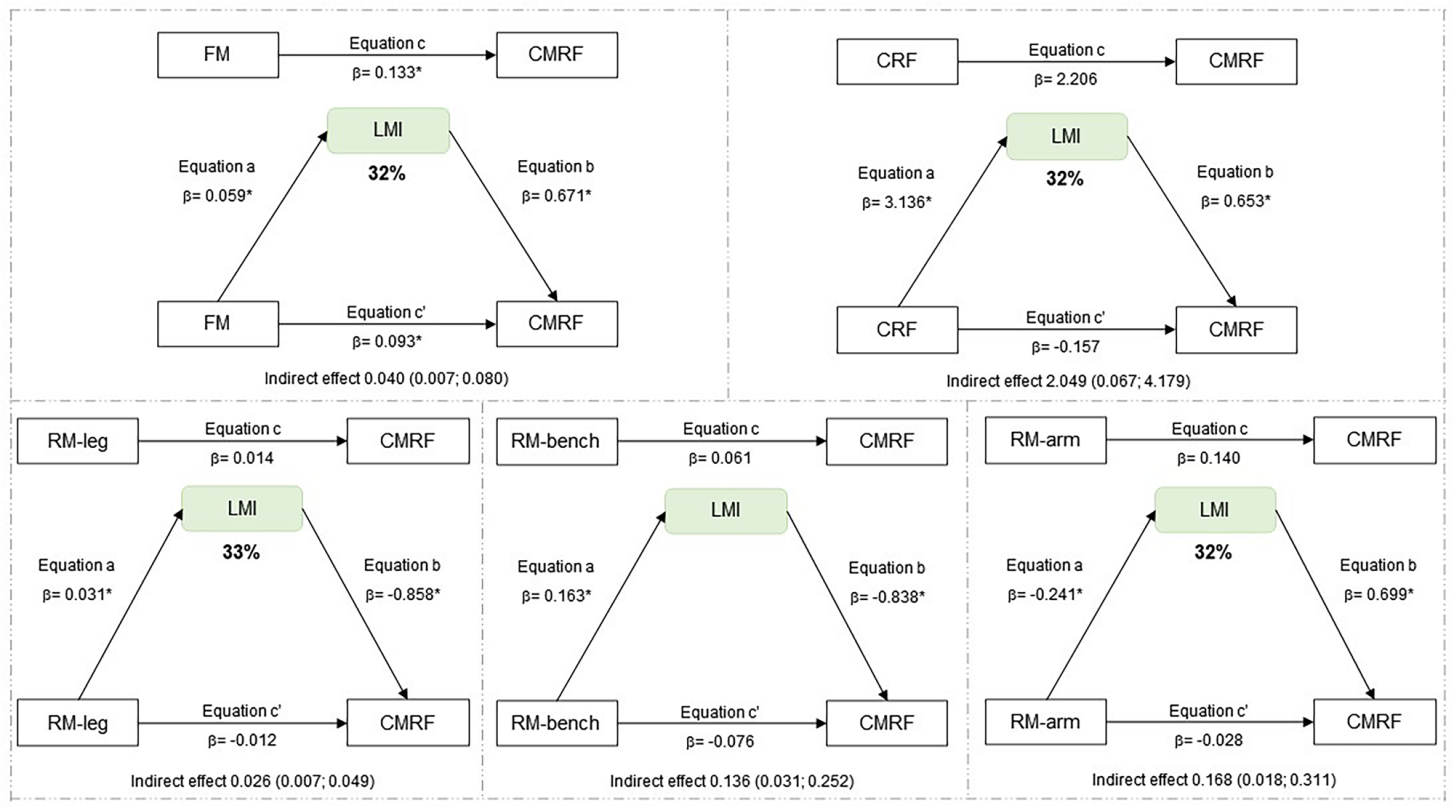
Mediation of lean mass index in the relationship between physical fitness and cardiometabolic risk in girls. Analyzes were controlled by age, age at peak height velocity and fat mass. *CRF* cardiorespiratory fitness, *RM-leg* repetition maximum-leg press, *RM-bench* repetition maximum-bench press, *RM-arm* repetition maximum-arm curl, *LMI* lean mass index, *CMRF* cardiometabolic risk factors score, *Significance p ≤ 0.05.

**Figure 4:**
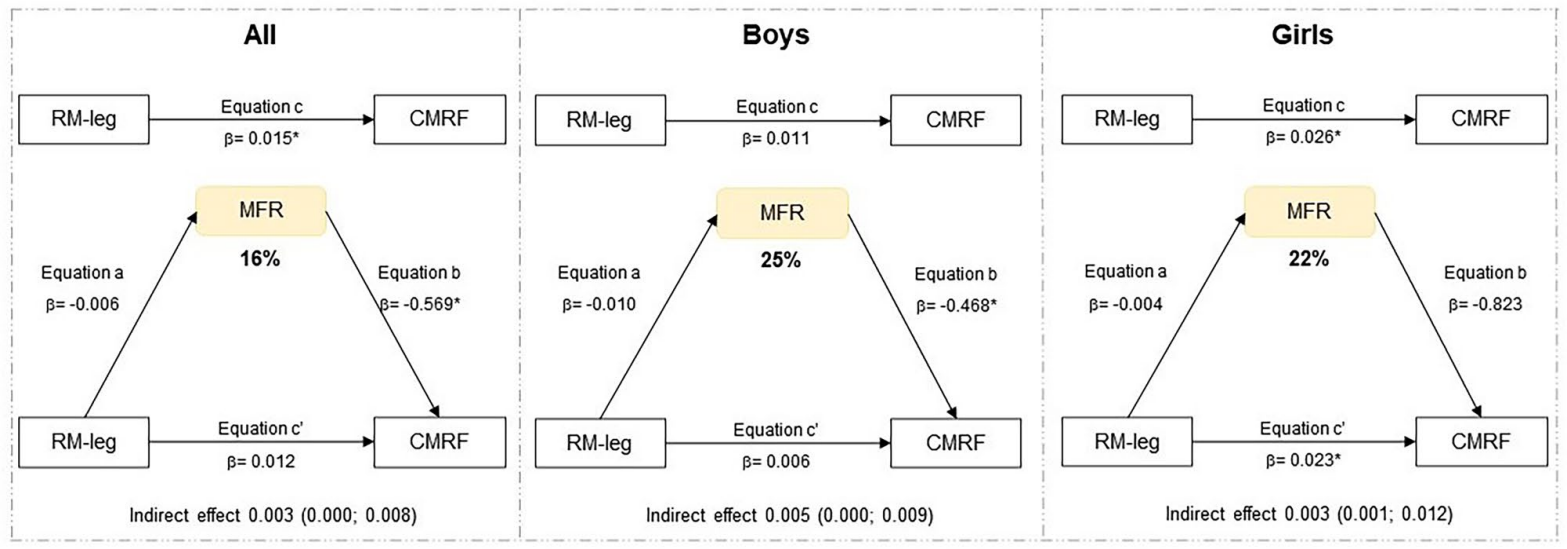
Mediation of muscle-to-fat ratio in the relationship between muscular fitness and cardiometabolic risk separated into all, boys, and girls. Analyzes were controlled by age and age at peak height velocity. *RM-leg* repetition maximum-leg press, *MFR* muscle-to-fat ratio, *CMRF* cardiometabolic risk factors score, *Significance p ≤ 0.05.

Regarding the mediation analyses, the indirect effect was significant, indicating LMI mediation in the relationship between fat mass and CMRF, as well as between CRF and CMRF for the total sample (Fig. 1). In the analyses of boys, the indirect effect was not significant, indicating that LMI is not a mediator in the relationship between fat mass, CRF, RM with CMRF (Fig. 2). In the analyses of girls, LMI mediation in the relationship between fat mass and CMRF, as well as between CRF, RM-leg press, RM-arm curl and CMRF (Fig. 3). MFR mediation in the direct relationship between RM-leg press and CMRF (Fig. 4).

## Discussion

In this study, the main findings indicate that muscle quantity shows a 26% mediation between CRF and CMRF in the total sample, whose relationship is most evident for girls (32%). LMI mediates 32% between fat mass and CRMF. Regarding MFR, a 16% mediation effect was observed between RM-leg press and CMRF, being found as 25% in boys and 22% in girls. However, the other indicators of muscle quantity assessed by the appendicular skeletal results, both ASM and ASMI showed no mediating effect in the analyses performed. Therefore, LMI and MFR seem to be better mediators of muscle quantity in adolescents between the variable’s adiposity, cardiorespiratory and muscular fitness with the CRMF. To the best of our knowledge, this is one of the first studies to examine the mediating role of muscle mass quantity in the relationship between physical fitness and cardiometabolic risk score in adolescents.

The importance of our study is emphasized, since there is an inverse relationship between cardiometabolic risk and low muscle mass or disproportionate muscle mass (in relation to fat)^15,16^. The disproportionate between of fat and lean mass was mainly analyzed in the context of adults, the elderly, and/or those with chronic diseases^15^. In the elderly, this relationship is confirmed by the amount of ASM (kg) divided by the squared height (m^−2^), while for adults, the diagnosis is made by the ratio of ASM (kg) divided by BMI (kg/m^−2^), whose adjustment is intended to reduce the interference that weight may have on lean mass^30^. For youth, proposals for verifying muscle quality include the ratio of muscle mass and fat mass or MFR^41^, handgrip^42,43^ and LMI^31^. Thus, Ripka et al.^31^ conducted a study that established curves with percentiles for fat mass, lean mass, LMI, ASM by DXA for Brazilian adolescents. They proposed LMI values of the 3rd percentile as the lower limit, aiming to identify LM abnormalities in the 12- to 17-year-old age group, which can be used for the diagnosis of decreased lean mass and relate to cardiometabolic risk, especially in overweight adolescents. Thus, excess fat mass in relation to muscle mass may be associated with low muscle mass, by reduced strength (i.e. dyapenia), as well as reduced muscle function related to cardiometabolic factors, which usually accompany obesity.

In the present research, in the comparison between adiposity groups by BMI-z, we confirmed that body weight overload exerted daily muscle work in adolescents with obesity, demonstrated by higher LM and LMI, as well as higher RM-leg press compared to eutrophic and overweight adolescents. While the ASM and ASMI did not differ between the groups, denoting no discrimination of muscle quantity in adolescents. However, it is reinforced that we did not evidence the presence of disproportion between fat and lean mass in adolescents with obesity in the current study, whose LMI values were above the 3rd percentile^31^ and adolescents with obesity showed CRF similar to eutrophic adolescents, in which muscle quantity, represented by LMI and MFR, played a mediating role in the reduction of CMRF.

In other studies, adolescents with obesity also demonstrated greater absolute muscle strength compared to their non-obese peers, but showed low muscle strength relative to body mass, low muscle fitness, and reduced neuromuscular activation capacity^14,16,44^. Adolescents with obesity can compensate for their greater body weight by increasing the level of voluntary activation during muscle contraction^45^, which may reflect in positive adaptation of the neuromuscular system to weight-bearing tasks. However, adolescents with obesity exhibit higher energy expenditure in performing body movement and increased muscle fatigue rate during exercise, partially explaining their limited involvement in physical activities, reduced muscle fitness and mobility^46^. Therefore, they may have difficulty maintaining muscle strength due to being overweight and possibly cause muscle weakness, which in turn may be caused by reduced mobility, neural adaptations, and changes in muscle morphology.

In our study, in the analysis of the MFR we noticed that it was higher in eutrophic, whose indexes were followed in a decreasing manner by the overweight and then in the obese, because the advantage of the higher LM may present progressive impairment caused by the higher fat mass. However, adolescents with obesity showed no disadvantages in cardiorespiratory and muscular fitness, despite showing impairment in most of the mean cardiometabolic variables. Expected cardiometabolic outcomes, as overweight and obese children and adolescents are more likely to have early cardiometabolic risk factors, such as high blood pressure^47^, disorders in lipid metabolism^47^, in glucose metabolism^48^ and in the inflammatory process^47^. This can lead to the development of chronic diseases such as type 2 diabetes mellitus, non-alcoholic fatty liver disease, endothelial dysfunction, and musculoskeletal dysfunction in adulthood^49,50^.

Recent evidence has shown that dyapenia and muscle mass contribute to adverse cardiometabolic health outcomes in children and adolescents^51^. In Korean adolescents, low skeletal muscle mass was associated with metabolic syndrome, and even after controlling for confounding factors, the risk of metabolic syndrome was higher in adolescents with low muscle mass^49^. An observational study found that adolescents at the 25th percentile of muscle mass had higher waist circumference, blood pressure, triglycerides, TC/HDL-c, HOMA-IR, and metabolic syndrome z-score than their peers at other percentiles, as well as adolescents with low muscle mass and obesity had the most unfavorable cardiometabolic profile^52^.

Research suggests that a body composition phenotype, which combines high-fat mass and low muscle mass, may be associated with higher health risks in adults, compared to any one body composition compartment alone^53,54^. The underlying mechanisms by which obesity affects skeletal muscle remain poorly understood; however, lipid accumulation in skeletal muscle and chronic low-grade inflammation may contribute to muscle impairment^44^. The positive energy balance leads to excessive fat accumulation in the subcutaneous region and between skeletal muscle fibers^55^. Fat accumulation initiates a systemic inflammatory response characterized by infiltration of immune cells into skeletal muscle tissue and increased secretion and activation of pro-inflammatory cytokines by myocytes and adipocytes. Because of chronic exposure to pro-inflammatory cytokines, satellite cell function may be affected, as well as myoblast proliferation and differentiation, negatively impacting skeletal muscle maintenance and regeneration^56^.

In addition to muscle mass, muscle strength is also an important aspect of physical fitness^14,44^, a protective factor of physical and cardiometabolic health both in the adult and elderly population^8^ and in children and adolescents^16,23^. These studies suggest that both low muscle mass and low muscle strength are contributors to metabolic dysfunction in the pediatric population^25^, as well as early onset of hypertensive measures^26^. Recent evidence focuses on reduction in muscle mass and cardiometabolic changes^16,23^, as well as understanding the mediating role of muscle quantity in the relationship between adiposity and bone mineral content^27^. Furthermore, although the interaction between reduced muscle mass and associated morbidities have not been investigated in detail in children and adolescents, excess fat with low muscle mass probably arises in childhood, reducing mobility and agility in children and adolescents^12^, and consequently compromising motor competence in adolescents with obesity^11^ and metabolic health before adulthood^57^.

This study has some strengths and limitations. The strengths were to assess body composition by DXA, cardiorespiratory fitness by stress test with metabolic analyzer, and muscular fitness by the one repetition maximum test, considered the best methods for assessing physical fitness. We used some variables to compose a cardiometabolic risk score and were performed by valid and reliable methods for monitoring the adolescent’s health. Also, adolescence is a period of somatic and biological growth, and changes can confound the results, our analyses were controlled for sex, age, and somatic maturation (age at peak height velocity). Finally, as far as we know, this study may be the first to consider LMI as a mediator in the relationship between physical fitness and a relevant marker of cardiometabolic health. On the other hand, we consider the cross-sectional design as one of the limitations, for not allowing the inference of causality, as well as the sample size used does not allow the generalization of the results, factors that should be analyzed with caution.

The results found in this study provide data on the association of muscle quantity, physical fitness, and cardiometabolic risk in adolescents, factors that contribute to the prevention and maintenance of health in this population. We highlight that LMI was identified as a mediator of muscle quantity in the relationship between fat mass vs. CMRF in total adolescents, plus CRF vs. CMRF in girls, who generally have lower participation in regular physical activity. While in boys there was no significant relationship between adiposity, physical fitness and CRMF. In addition, the MFR showed a mediating role in the relationship between RM-leg press and CMRF. Therefore, we highlight the importance of muscle quantity as a mediator in physical fitness and fat mass, for the reduction of cardiometabolic risk factors, justifying the importance of a healthy lifestyle in children and adolescents, especially with emphasis on girls.

## Data Availability

Data are available on request from the authors. Requests can be sent to the corresponding author Maiara Cristina Tadiotto at the e-mail address mctadiotto@gmail.com.
